# Behavioral and Endocrine Alterations to Partner Interactions and Offspring Care during Periods of Conflict

**DOI:** 10.1093/iob/obaa002

**Published:** 2020-01-28

**Authors:** Timothy Paciorek, Leese Joseph

**Affiliations:** 1 Department of Biological Sciences, tpp312@lehigh.edu Lehigh University, Bethlehem, PA 18015, USA; 2 Department of Biology, DeSales University, Center Valley, PA 18034, USA

## Abstract

Biparental care has evolved to ensure successful rearing of offspring. However, separation during periods of care can lead to conflicts that might negatively impact pair bonds and offspring care. In this study, pair-bonded convict cichlids (*Amatitlania nigrofasciata*) were observed for changes in behavior toward their partners and offspring before and after a period of separation. Males and females were designated either as a Resident (remain with offspring) or Removed (separated from partner and offspring for 5 days) individual. Behaviors between partners and toward offspring were measured before and after separation, and compared to the levels of behavior of control pairs (never separated), as well as individuals introduced to a novel partner instead. Cortisol levels of Resident male and female *A. nigrofasciata* were assayed using water-borne hormone collection before and after separation. Aggression between pair bond members did increase following reintroduction, but did not lead to the termination of pair bonds. Resident females showed more aggression to novel partners than Resident males. Offspring care decreased in both Resident and Removed females. Experimental pairs decreased the amount of time spent interacting with intruders. Cortisol levels were significantly higher among experimental pairs compared with control pairs that did not experience a separation. Females (both control and experimental) showed small, yet significant increases in cortisol levels, while both control and experimental males did not. These results suggest that while pair bonds appear resilient, prolonged separations influence pair bond and parental care dynamics, both behaviorally and hormonally, and require pairs to re-establish roles, resulting in less time caring for offspring.

## Introduction

Monogamy functions as a mating strategy in which both parents provide care for offspring through behaviors such as feeding (Leffellaar and Robertson 1986), defense (van Breukelen and Itzkowitz 2011), and cultural transmission of information (Al-Shaer et al. 2016). Whereas many species engage in parental care that is limited to one parent, monogamous strategies are thought to provide a fitness advantage to parents through the establishment of biparental care (for review, see Kvarnemo 2018), as combined participation in the rearing of offspring should ultimately lead to increased offspring survival (Clutton-Brock 1991).

Despite the benefits of monogamous breeding systems (Kvarnemo 2018), there are fitness costs associated with each participant’s investment, as extended offspring care may limit future breeding opportunities (Trivers 1972). If an individual reduces its parental investment so as to increase its chances of future reproductive success, then the monogamous pair bond may be placed at risk. Individuals that have been abandoned are known to adopt their partner’s roles in order to care for offspring, with both males and females demonstrating the ability to take on the roles of their missing partners when separated (Sasvári 1986; Wynne-Edwards 1987; Bart and Tornes 1989; Lavery and Reebs 1994; Markman et al. 1996; Lehtonen et al. 2011; van Breukelen and Itzkowitz 2011; Snekser and Itzkowitz 2014). This research has often led to the conclusion that single parents are inefficient at caring for offspring to the point of reduced offspring survival, and thus reducing their own reproductive success. Males are thought to be more likely to abandon pair bonds since they are only limited by numbers of available spawning opportunities, unlike females that are limited more by energy and time required to produce eggs which limits their reproductive success (Trivers 1972).

Changes in social cues and environmental stimuli may also influence hormonal state. This is important within parental care behaviors as there are often a multitude of hormones at play that regulate how parents respond to the needs of offspring. A large proportion of research into the links between hormones and behaviors has been conducted in birds. Lehrman (1965) observed that hormone concentrations of estradiol, progesterone, and prolactin in ring doves were correlated with mutually exclusive behaviors during courtship, nest building, and parental care. These behaviors were disrupted when pairs were separated from one another or when courtship and parental care activities were presented out of order, likely influencing the release of steroid hormones and prolactin involved in extended parental care. However, exogenous administration of estradiol, progesterone, and prolactin was shown to restore the suite of behaviors involved in courtship and parental care. Excessive hormone concentrations can also have detrimental effects on parental care as demonstrated by Hegner and Wingfield (1987), who observed that male house sparrows treated with exogenous testosterone showed decreased offspring care, indicating that testosterone is more involved in territorial defense and courtship rather than parental care behaviors. Mammals, including rodents, also show correlations between parental behavior and hormonal state, with ovariectomized, nulliparous females engaging in parental care when given exogenous hormones including estradiol, progesterone, and prolactin (Moltz et al. 1970).

The influence of hormones in parental care has also received attention in monogamous cichlid species. Male cichlids that have fathered offspring show decreased levels of testosterone and 11-ketotestosterone (11-KT; Earley and Hsu 2008). The formation of pair bonds in cichlids are also believed to be influenced by nonapeptides such as vasotocin (AVT: non-mammalian homolog of vasopressin) and isotocin (IT: non-mammalian homolog of oxytocin) (Oldfield and Hofmann 2011). In particular, isotocin antagonists have been shown to reduce parental care in males, further supporting the notion of the complexity of this system and its endocrine state (O’Connell et al. 2012). One likely possibility is that changes in behavior lead to increased glucocorticoid secretion, which in turn alters the expression of isotocin, vasotocin, and nonapeptide receptors. However, compared with sex steroids, nonapeptides are not as stable in water and therefore are not as easily extractable.

Glucocorticoids such as cortisol are viewed as a primary indicator of elevated stress (Wong et al. 2008; Gabor et al. 2013). Cortisol is a cholesterol-derived steroid hormone that is known to play a vital role in a variety of biological processes including homeostasis, metabolic resource partitioning, and other adaptive responses to danger (Mommsen et al. 1999). Cortisol is believed to be beneficial on an acute level, however, long-term secretion of the hormone has been linked to detrimental health effects, particularly with regard to reproduction (McEwen 1998). In rats, increases in plasma glucocorticoid levels were shown to intensify aggression between individuals (Mikics et al. 2004). In staged contests, fluctuations in levels of circulating glucocorticoids, as well as androgens like testosterone, are correlated with bouts of aggression (for review, see Hsu et al. 2005). Social interactions, like maintaining a pair bond or caring for offspring, are fraught with potential stressors that may impact an individual’s response (Siegel 1980; Keay et al. 2006; Wong et al. 2008).

Pair bond maintenance involves multiple stressors that ultimately could result in one individual leaving its partner. Whereas many studies have examined how partners function when left as single parent, it is still unclear how conflicts affect pair bonded individuals when they are reunited with partners that abandoned them. If pair bonds have terminated due to conflict over parental investment, then it is possible that upon reuniting there will be differences not only in behaviors concerning the welfare of offspring, but also in responses to partners themselves. Single parents must invest more in care for offspring, though often not as efficiently as intact pairs (Wynne-Edwards 1987; Bart and Tornes 1989; Lavery and Reebs 1994; Snekser and Itzkowitz 2014). Mackie and Itzkowitz (1985) observed that separation and reintroduction of Texas cichlids (*Cichlasoma cyanoguttatum*) during parental care was successful based on time, with pairs more likely to reunite after a single day, while separations of 4–10 days resulted in a 50–100% breakdown of the pair bond. Furthermore, it is unclear whether both males and females will attempt to resolve conflict by reforming a pair, though theoretical work has indicated that conflicts over parental investment may be accompanied by periods of negotiation in which both parents allocate how much care they are prepared to provide (Houston and Davies 1995; McNamara et al. 1999; Johnstone and Hinde 2006). These models allowing both parents to make continuous decisions about the amount of parental investment they should provide, which in turn may lead to more benefits to raising offspring.

The convict cichlid (*Amatitlania nigrofasciata*) provides a unique opportunity to examine factors that may influence pair bonds. This species forms monogamous pair bonds and engages in extended biparental care of young (∼4–6 weeks) with a division of parental sex roles (Wisenden 1995). Males typically engage in territory defense while females stay in close proximity to offspring, though both parents are known to switch roles (Snekser and Itzkowitz 2009). The aim of this study was to determine whether male and female *A. nigrofasciata* respond to abandonment and reintroduction into pair bonds similarly, and whether changes in behavior are present that will lead to decreased parental care and dissolving of pair bonds. Two experiments were established to address this. The first experiment determined whether disruptions to pair bonds caused changes in behavior between partners. Three hypotheses were addressed. First, it was hypothesized that abandoned *A. nigrofasciata* (both males and females) would show increased aggression when introduced to novel partners compared with previous partners, as they would not be familiar with this individual and view them as a threat to their offspring. Second, it was hypothesized that following periods of separation, aggression would increase among monogamous pairs, with abandoned partners less likely to incorporate their former partner back into a pair bond compared with an intact pair. Specifically, females who were abandoned by their mates were expected to show the highest levels of aggression, as they are more likely to experience this phenomenon in nature and suffer more costs to offspring care compared with males. Finally, it was hypothesized that parental care behaviors, in the form of direct offspring care and territory defense which are the two primary care behaviors for *A. nigrofasciata*, would increase in the abandoned partner following reintroduction to their partner, as any period of conflict resolution may require them to spend more time performing their partner’s sex specific role until pair bonds are reestablished. The second experiment examined changes in circulating cortisol levels in the abandoned fish before and after partners were removed and reintroduced. It was hypothesized that parental conflict increases the secretion of cortisol, and that cortisol acts to inhibit pair bonding and decrease parental care. It was predicted that both males and females abandoned by their partners would show increased levels of cortisol when reintroduced to their partners in relation to attempting to reincorporate their partners into the pair bond and their ability to engage in parental care activities.

## Methods

### Fish stock and housing

Prior to experimentation, all adult *A. nigrofasciata* were housed in single-sex 473-l stock aquaria. All *A. nigrofasciata* used in the study were either bred in the laboratory or purchased from commercial suppliers with captive stock. All fish were fed commercial pellet food daily (Ken’s premium sinking krill pellets) and kept at ∼27 °C on a 14 L:10 D photoperiod. Fish acquired from outside suppliers were held in the laboratory for approximately 3 months prior to being used in the study.

## Behavioral study

### Experimental setup

Male and female *A. nigrofasciata* were selected haphazardly from stock aquaria. Using calipers, the total length (TL) of each fish was measured before being placed into mating pairs. Male *A. nigrofasciata* were approximately 10.0 ± 5.0 mm larger than females (mean TL ± SD: experimental males 80.4 ± 8.3 mm, experimental females 69.7 ± 8.2 mm, control males 79.8 ± 7.5 mm, control females 68.9 ± 6.8 mm, novel partner males 81.2 ± 9 mm, novel partner females 69.8 ± 8.7 mm, novel males 82.1 ± 6 mm, and novel females 67.9 ± 8.3 mm) in order to facilitate pair bond formation. This is due to natural female preference toward larger males (Wisenden 1995; Gagliardi-Seeley et al. 2009). TL did not significantly differ among treatment groups for males (ANOVA: *F*_3,123_ = 0.323, *P* = 0.809) or females (ANOVA: *F*_3,126_ = 0.260, *P* = 0.854). Each pair was placed into a 75-L tank with a thin layer of gravel and an aeration stone. A small clay pot (11 cm in diameter) was placed in the corner of the tank as a shelter and substrate for egg deposition.

#### Part 1: Examination of pair bonds before separation

Each *A. nigrofasciata* pair was allowed 2 weeks to form a pair bond and lay eggs. Clay pot shelters were examined daily for the presence of eggs. Pairs that did not lay eggs were removed from the experiment and placed into new single sex stock tanks that were not sampled during the course of the experiment. Pairs were randomly assigned to one of three groups: control, experimental, or novel partner. Control pairs (*N* = 40) remained intact for the duration of the experiment, but were randomly assigned to have either the male (*N* = 20) or female (*N* = 20) go through a sham removal. Experimental pairs (*N* = 48) had either the male (*N* = 24) or female (*N* = 24) member of the pair bond removed from its partner for 5 days and then reintroduced. Novel partner pairs (*N* = 27) had either the male (*N* = 15) or female (*N* = 12) member of the pair bond removed and replaced with a novel male or female following the 5-day separation period.

Eggs hatched in approximately 3 days. Newly hatched *A. nigrofasciata* remain in a wriggler stage for approximately 5 days where they are semi-immobile due to continued development post-hatching. The experiments described here took place during the duration of the wriggler stage so as to keep track of offspring location more easily than with free-swimming fry. Once wrigglers were present, *A. nigrofasciata* pairs were filmed continuously for 30 min using high-definition JVC cameras. Recordings began when a small juvenile *A. nigrofasciata* was placed in a Plexiglas cage (91 cm^2^) approximately 38 cm from the clay pot. Juveniles were selected haphazardly from a separate 189-l stock tank (mean TL = 48.3 mm). During the recordings, male and female *A. nigrofasciata* were examined for two parental care behaviors: time spent with wrigglers and time spent defending territories from the juvenile *A. nigrofasciata*. Juvenile *A. nigrofasciata* are common predators of wrigglers and were used in order to elicit territory defense from parents. Pairs were also examined for aggressive behavior toward one another. Three aggressive behaviors (bites, chases, and gill flares) that males and females displayed toward one another were recorded. Aggressive behaviors were pooled to form one category of “total aggression” for data analysis.

#### Part 2: Examination of pair bonds after reintroduction

Following the first recording, pair bond members were assigned to be either the “Resident” fish (stays with offspring) or the “Removed” fish (removed from its partner and offspring for 5 days). This was done in order to simulate pair bond abandonment. For experimental groups, the male or female removed fish was netted from the tank and placed into a separate, identical 75-l tank for 5 days. Plastic opaque barriers were placed in between adjacent tanks so that fish could not see one another. In control groups, the removed male or female was subjected to a sham removal, where they were netted for approximately 10 s before being placed immediately back in the tank with its resident partner for 5 days. In novel partner groups, the removed males and females were placed into separate single stock aquaria so as not to be re-used. Following the 5-day separation period, a novel male or female (within approximately 5 mm of the original partner) was introduced to the resident fish and offspring. It could not be determined whether novel partners had prior breeding experience. A 5-min acclimation period was given prior to the start of recording. Tanks were recorded identically to Part 1, with parental care and aggressive behaviors monitored. Following completion of the experiment, adult *A. nigrofasciata* were placed into single sex stock aquaria separate from those sampled during the experiment. Wrigglers were placed into separate stock aquaria.

## Endocrine study

### Experimental setup

New male and female *A. nigrofasciata* were sampled and measured identically as was performed in the behavioral study (mean TL ± SD: experimental males 76.8 ± 5.8 mm, experimental females 66.4 ± 6.3 mm, control males 79.4 ± 8.1 mm, control females 67.7 ± 6.4 mm). No fish from the behavioral study were used in these experiments. TL did not significantly differ among treatment groups for males (*t*_38_ = 1.161, *P* = 0.253) or females (*t*_38_ = 0.666, *P* = 0.510). Pairs were given identical housing to ones from the behavioral study.

Pair bond formation followed the same procedures as stated in the behavioral study. Pairs were randomly assigned to one of two groups: control or experimental. Control pairs (*N* = 16) remained intact for the duration of the experiment, but were randomly assigned to have either the male (*N* = 8) or female (*N* = 8) go through a sham removal. Experimental pairs (*N* = 24) had either the male (*N* = 12) or female (*N* = 12) member of the pair bond removed from its partner for 5 days before being reintroduced. Pairs were recorded in a similar fashion to the behavioral study in order to maintain consistency in design parameters. However, no behavioral data was used in subsequent analyses.

#### Part 1: Habituation to beaker confinement

Prior to the start of the experiment, resident males and females were subjected to three consecutive days of 30-min beaker confinement. This coincided with the presence of eggs so that once habituation was completed, the eggs would have hatched into wrigglers to begin the experiment. Individuals were transferred from their test tanks to a clean 600 mL beaker filled with 300 mL of clean water for 30 min in order to obtain naturally secreted hormones. All beakers were cleaned with 100% ethanol and distilled water prior to collections. This collection technique and time has been used successfully across a variety of aquatic species (Sebire et al. 2007; Wong et al. 2008). Collection occurred between 09:00 and 11:00 h.

#### Part 2: Hormone extraction, processing, and assay

Immediately following each recording, resident males and females were transferred from their test tanks to a clean 600 mL beaker filled with 300 mL of clean water for 30 min in order to obtain naturally secreted hormones. Once the hormone collection process was complete, the contents of the beaker were poured through a net washed in distilled water and into 500 mL glass Kimax bottles. All bottles were washed with 100% ethanol and distilled water prior to the start of experiments. Water samples were frozen at −20°C until processed.

Water samples were thawed at room temperature and filtered using Whatman Filter paper 1, and passed through Sep-Pack^®^ Plus C18 solid phase extraction columns (2 mL) (for methods, see Earley et al. 2006). Columns were primed using 2 × 2 mL methanol (MeOH) followed by 2 × 2 mL distilled water. Samples were passed through Saint-Gobain Tygon^®^ tubing (washed with 100% ethanol and distilled water) and into the columns using a vacuum manifold.

Once water samples were passed through the columns, they were eluted into 12 × 75 borosilicate vials using ethyl acetate to isolate free circulating cortisol from conjugate cortisol (for review, see Ellis et al. 2004). Eluted samples were immediately evaporated under a gentle stream of nitrogen in a water bath at 37°C. Evaporation results in a residue of hormones that was re-suspended in distilled water and ethanol. Samples were further re-suspended in EIA buffer provided by commercially available Cortisol EIA kits (Cayman Chemicals).

A pooled *A. nigrofasciata* water-borne hormone extract from all 80 samples was used in order to validate the EIA kit and subsequent dilutions used. The pooled sample was serially diluted (1:1–1:128) and the slope of the dilution curve compared with the curve created by standards within the EIA kit. A dilution of 1:2 was used for each sample based on the standard curve. A total of four 96-well plates were assayed for each group with all samples run in triplicate. The inter-assay co-efficient of variance for male control, female control, male experimental, and female experimental were 58.4%, 27.1%, 47.3%, and 60.2%, respectively.

### Statistical analyses

Data were analyzed using IBM SPSS Statistics version 23. Comparisons of control, experimental, and novel partner total aggression were examined during the second recording only, as the novel partner residents only interacted with the novel individual once, therefore making it impossible to do a comparison over time, and therefore were analyzed using a univariate one-way ANOVA. Pairwise comparisons of groups were performed using Bonferroni *post* *hoc* analyses. In order to determine if there were differences in aggression and parental care behaviors before and after each separation period, data were analyzed using mixed-design ANOVAs. Total aggression, time with offspring, and time with intruder served as the dependent variables, with time (i.e., “Before” and “After”) serving as within-subject factors, and treatment group (control and experimental), status (resident and removed), and sex (male and female) served as between-subject factors. Pairwise comparisons of interactions between factors were performed using Bonferroni *post* *hoc* analyses.

For Experiment 1, five pairs of data points (one from experimental removed females, one from experimental resident males, one from experimental resident females, one from experimental removed males, and one from control resident females) for total aggression were identified as outliers. This was based on examinations of skew and kurtosis when establishing the normality of the dataset. When using a skew value of ±2 and a kurtosis value of ±7 (Byrne 2010; Hair et al. 2010), total aggression data were found to be heavily skewed, and thus these data points were subsequently removed from the analyses, which improved the distribution of the data.

For Experiment 2, in order to determine differences in cortisol levels in resident fish before and after each separation period, data were analyzed using mixed-design ANOVAs. Cortisol levels (pg/mL) served as the dependent variable, while time (i.e., “Before” and “After”) served as the within-subjects factor, while treatment group (control and experimental) and sex (male and female) served as the between-subject factors. Pairwise comparisons of interactions between factors were performed using Bonferroni *post* *hoc* analyses. With the exception of the experimental resident females, a single pair of outliers was identified in the control resident male and control resident female groups, and two outliers were found in the experimental resident male group, which were subsequently removed from analyses using the same skew and kurtosis criteria used in Experiment 1 (Hair et al. 2010; Bryne 2010).

## Results

### Behavioral study

#### Males and females differ in partner treatment

**Fig. 1:**
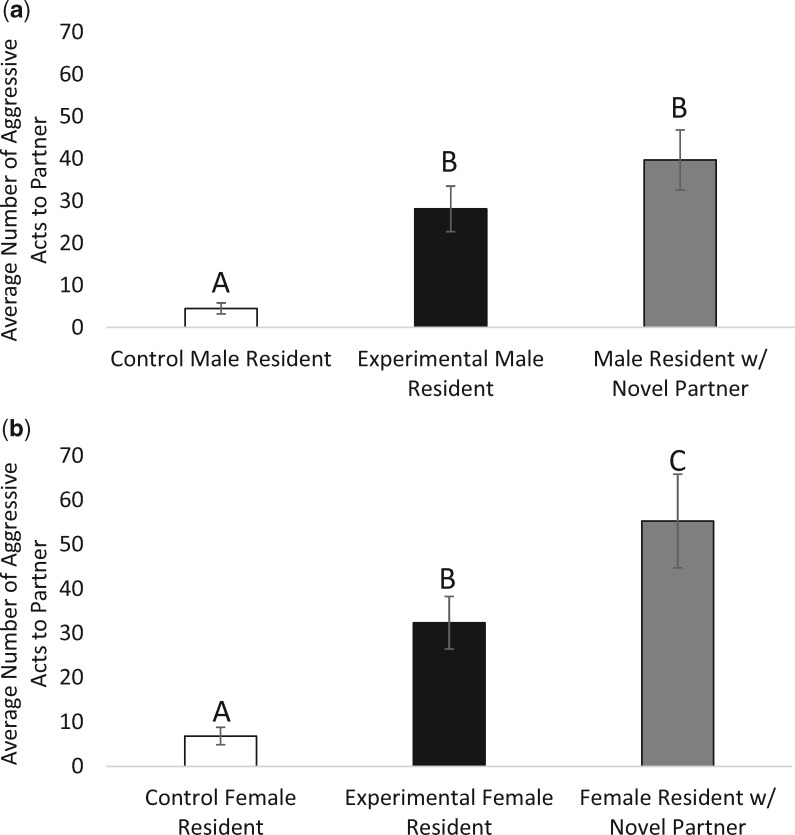
(**a**, **b**) Aggressive acts (±SE) displayed by resident males (**a**) and resident females (**b**). **a**) Experimental male residents showed no difference in aggression to their former partners compared with male residents that received a novel partner following separation. Both experimental groups showed significantly more aggression to partners compared with control male residents. **b**) Experimental female residents showed significantly less aggression to their former partners compared with female residents that received a novel partner following separation. Both experimental groups showed significantly more aggression to partners compared with control female residents. Different letters indicate significance between groups.

There was a significant difference in aggression displayed by resident males to female partners across all three groups (ANOVA: *F*_2,56_ = 12.003, *P* < 0.001, partial *ɳ*^2^ = 0.300) (Fig. 1a). Experimental male residents were shown to be more aggressive toward their returned partners compared with control male residents (*P* = 0.002), as were novel partner male residents compared with control male residents (*P* < 0.001). However, there was no difference in aggression when comparing experimental male residents to novel partner male residents after the separation period (*P* = 0.346).

Female residents showed a significant difference in aggression across groups (ANOVA: *F*_2,52_ = 13.113, *P* < 0.001, partial *ɳ*^2^ = 0.335) (Fig. 1b). Experimental female residents were more aggressive toward their returned partners than control female residents (*P* = 0.007), as were novel partner female residents compared with control female residents (*P* < 0.001). However, experimental female residents were significantly less aggressive toward their partners compared with novel partner female residents (*P* = 0.049).

#### Prolonged separation increased aggression between partners

**Fig. 2:**
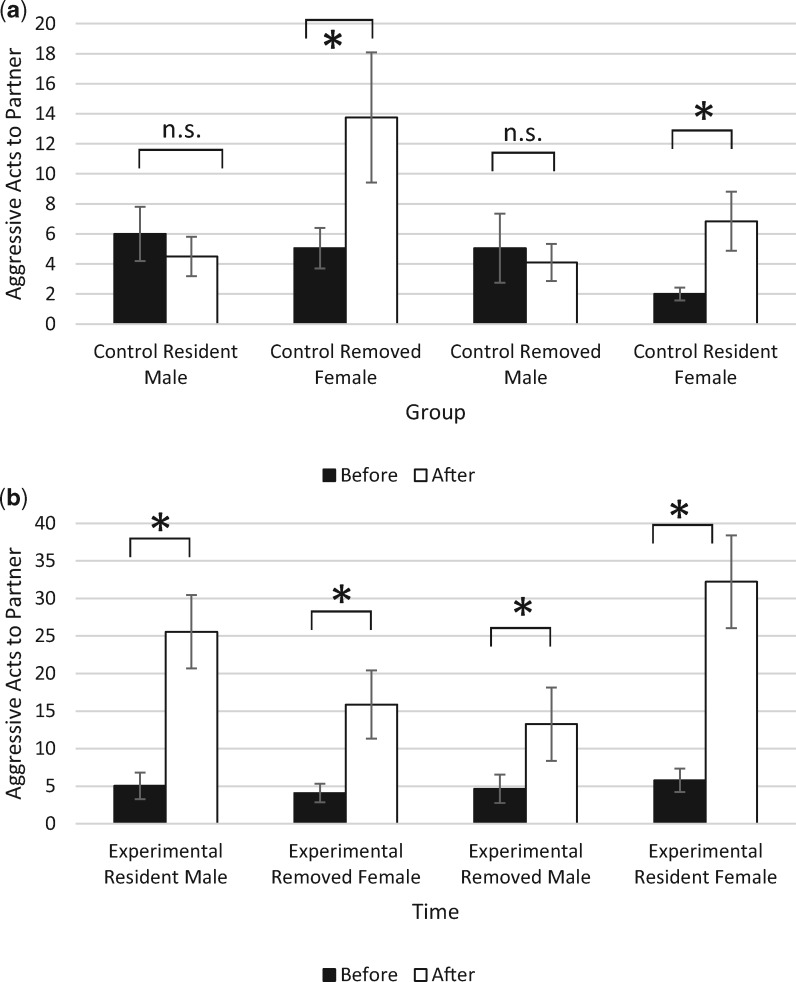
(**a**, **b**) Number of aggressive acts (±SE) displayed among males and females within control (top) and experimental (bottom) pairs before and after separations. **a**) Control females (both resident and removed) showed increases in aggression while control males did not. No individuals showed significant increases in aggression toward their partners. **b**) Both experimental resident and removed male and female showed increased aggression, with experimental resident females showing the greatest change in aggression among experimental and control fish. Asterisks indicate significance (*P* < 0.05) while n.s. denotes non-significance.

There was a main effect of time on total aggression (mixed ANOVA: *F*_1,163_ = 42.726, *P* < 0.001, partial *ɳ*^2^ = 0.208), with aggressive acts toward partners increasing over time. A main effect of treatment group was also observed (mixed ANOVA: *F*_1,163_ = 18.191, *P* < 0.001, partial *ɳ*^2^ = 0.430), with experimental pairs showing a more pronounced increase in aggression compared with control pairs. There was no main effect of sex (mixed ANOVA: *F*_1,163_ = 1.577, *P* = 0.211, partial *ɳ*^2^ = 0.010), or status (mixed ANOVA: *F*_1,163_ = 0.184, *P* = 0.668, partial *ɳ*^2^ = 0.001), indicating that regardless of their status, males and females did not differ in the number of aggressive acts performed. There was a significant four-way interaction between time, sex, treatment group, and status (mixed ANOVA: *F*_1,163_ = 6.662, *P* = 0.011, partial *ɳ*^2^ = 0.039) (Fig. 2a, b), indicating that experimental female residents displayed the greatest change in aggression compared with other fish.

Experimental female residents showed significant changes in aggression over time (*P* < 0.001), as did experimental removed females (*P* = 0.004). Experimental male residents also showed significant changes in aggression over time (*P* < 0.001), as did experimental removed males (*P* = 0.036). There were no significant changes in aggression for control resident females (*P* = 0.282), but control removed females did significantly increase their aggression (*P* = 0.048). Neither control resident males (*P* = 0.732) nor control removed males (*P* = 0.828) showed any change in aggression.

#### Offspring care is higher in females, but decreases over time

**Fig. 3:**
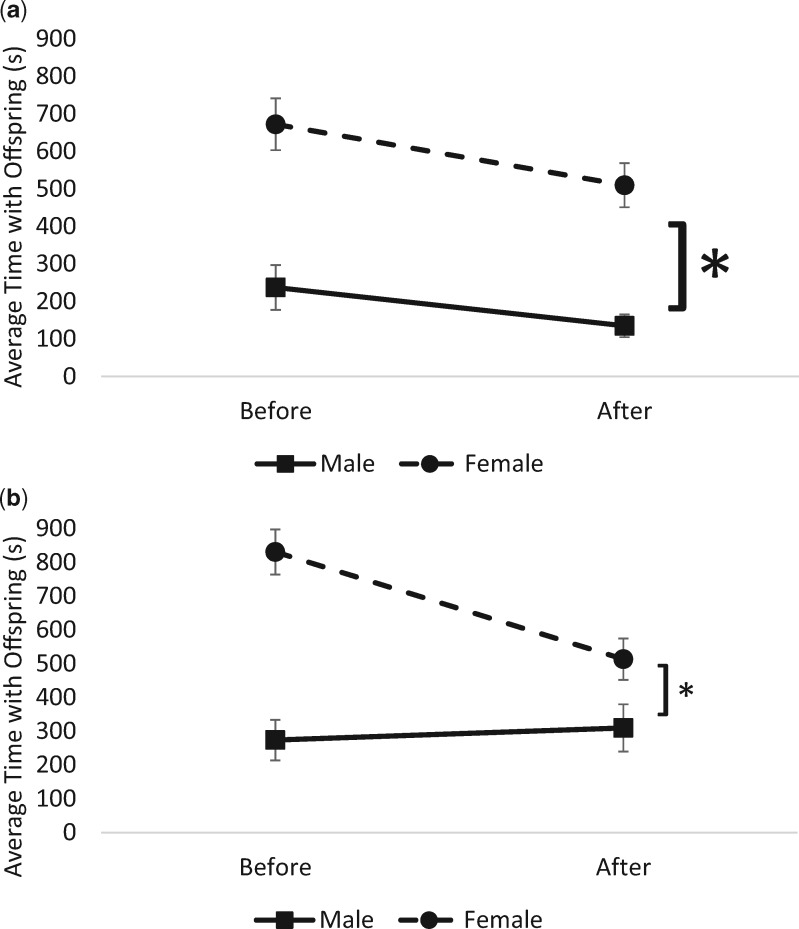
(**a**, **b**) Change in offspring interactions (±SE) among control (**a**) and experimental (**b**) males and females. **a**) Control females (both residents and removed) spent significantly more time with offspring compared with males. They showed a significant decrease in time spent with offspring following the return of their partner. **b**) Experimental females (both residents and removed) spent significantly more time with offspring compared with males. They showed a significant decrease in time spent with offspring following the return of their partner. Asterisks indicate significance (*P* < 0.05).

There was a significant main effect of time on offspring care (mixed ANOVA: *F*_1,68_ = 16.460, *P* < 0.001, partial *ɳ*^2^ = 0.089) and a significant main effect of sex (mixed ANOVA: *F*_1,168_ = 57.114, *P* < 0.001, partial *ɳ*^2^ = 0.254), indicating that females spent more time with offspring than males, though over the course of the experiment time with offspring decreased. There was a significant interaction of time, group, and sex on offspring care (mixed ANOVA: *F*_1,168_ = 4.724, *P* = 0.031, partial *ɳ*^2^ = 0.027) indicating that regardless of their status (resident or removed), experimental females significantly decreased the amount of time they spent with offspring (Fig. 3a, b).

#### Time with intruder decreases in experimental pairs

**Fig. 4:**
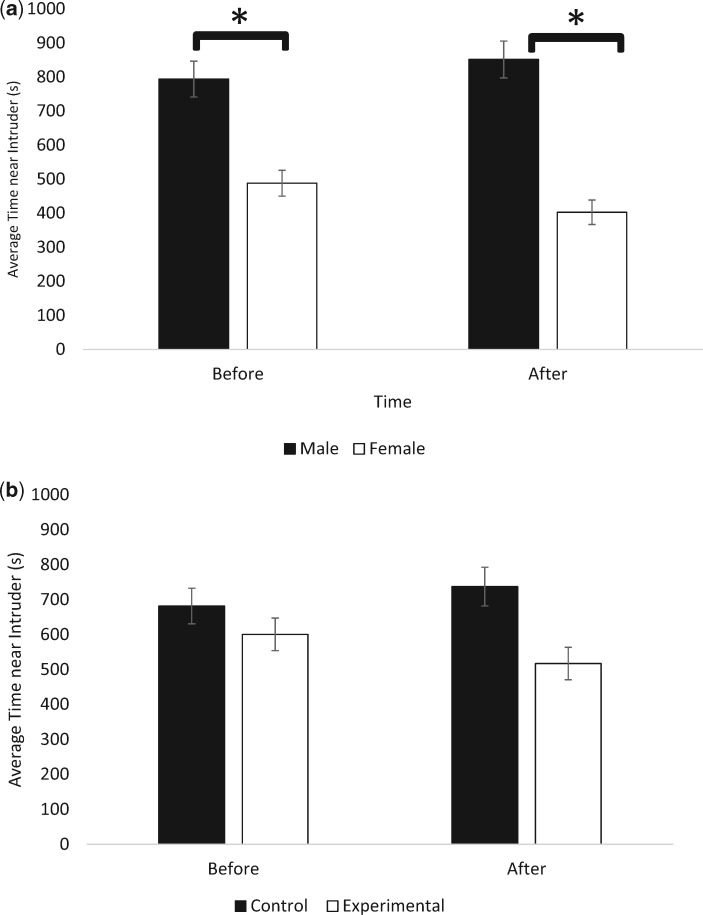
(**a**, **b**) Change in intruder interactions (±SE) based on sex (top) and between control and experimental pairs (bottom). **a**) Males spent significantly more time interacting with the intruder compared with Females. **b**) Experimental pairs spent significantly less time interacting with intruders than control pairs following partner reintroduction. Asterisks indicate significance (*P* < 0.05).

There was a main effect of sex on intruder interactions (mixed ANOVA: *F*_1,168_ = 47.081, *P* < 0.001, partial *ɳ*^2^ = 0.219), indicating that males spent more time interacting with the intruder compared with females. There was also a main effect of treatment group (mixed ANOVA: *F*_1,168_ = 7.512, *P* = 0.007, partial *ɳ*^2^ = 0.079), indicating that control pairs spent more time interacting with the intruder compared with experimental pairs. In contrast, there was no main effect of time (mixed ANOVA: *F*_1,168_ = 0.175, *P* = 0.676, partial *ɳ*^2^ = 0.001) or status (mixed ANOVA: *F*_1,168_ = 1.308, *P* = 0.246, partial *ɳ*^2^ = 0.008), indicating that time spent interacting with the intruder did not change following partner reintroduction, nor were there differences between resident and removed individuals with regard to intruder interactions. There was a significant interaction of time and treatment group on intruder interactions (mixed ANOVA: *F*_1,168_ = 4.328, *P* = 0.039, partial *ɳ*^2^ = 0.025), indicating that experimental pairs significantly decreased the amount of time spent interacting with the intruder following partner reintroduction compared with control pairs (Fig. 4).

## Endocrine study

### Experimental males show greatest increase in cortisol, while females show consistent increases in both groups

**Fig. 5:**
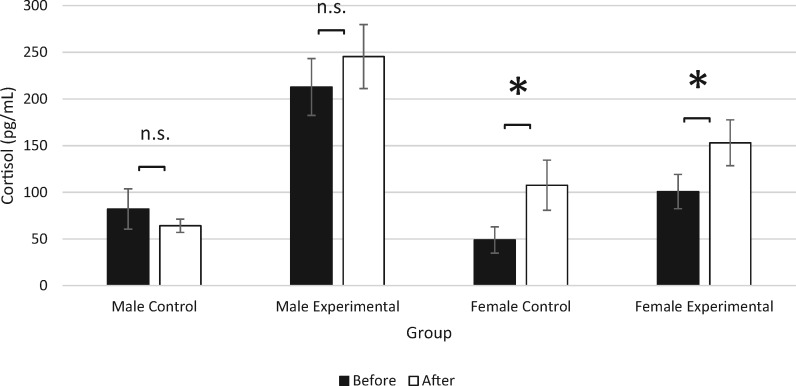
Change (±SE) in cortisol (pg/mL) in control and experimental male and female residents before and after separations. Control and experimental resident males showed no change in cortisol levels over time, while control and experimental resident females showed a significant increase in cortisol. Asterisks indicate significance (*P* < 0.05) while n.s. denotes non-significance.

There was no main effect of time on cortisol levels (mixed ANOVA: *F*_1,32_ = 3.399, *P* = 0.075, partial *ɳ*^2^ = 0.096), indicating that cortisol levels did not significantly increase over time. There were significant main effects of sex (mixed ANOVA: *F*_1,32_ = 5.957, *P* = 0.020, partial *ɳ*^2^ = 0.157), with males showing higher cortisol levels compared with females. Main effects of group were observed (mixed ANOVA: *F*_1,32_ = 26.451, *P* < 0.001, partial *ɳ*^2^ = 0.453), with experimental residents showing higher levels of cortisol compared with control residents. A significant interaction of sex and group was observed (mixed ANOVA: *F*_1,32_ = 7.271, *P* = 0.011, partial *ɳ*^2^ = 0.185), indicating that experimental males had significantly higher circulating levels of cortisol. There was no significant interaction of time, sex, and group (mixed ANOVA: *F*_1,32_ = 0.702, *P* = 0.408, partial *ɳ*^2^ = 0.021) (Fig. 5).

## Discussion

In this study, monogamous *A. nigrofasciata* pairs were observed for changes in their aggression toward one another as well as changes in parental care behaviors following the effects of partner separation. It was predicted that resident *A. nigrofasciata* (both males and females) would show increased aggression to novel partners compared with previous partners. Resident males showed a similar amount of aggression when introduced to a novel female and when re-introduced to their previous female partner. In contrast, resident females were nearly twice as aggressive toward a novel male than toward their previous male partner following re-introduction. Thus, the first hypothesis was not fully supported due to sex differences in aggressiveness. Differences in female aggression may indicate that they are more discerning when distinguishing former mates from novel individuals, possibly with regard to offspring safety. *Amatitlania nigrofasciata* have been shown to display differences in aggression based on sex, with female *A. nigrofasciata* tending to engage in more offspring defense against intruders entering monogamous pair territory (Wisenden et al. 2008). Aggressive signals may vary, in that novel individuals are treated more aggressively compared with former partners if they are considered a threat to offspring survival. Furthermore, if aggression is being displayed in part to reform the pair bond, this may explain why there are noticeable difference in aggression between males and females when they are introduced to novel partners. Female aggression has been suggested to control male access to offspring in order to facilitate sex-specific parental care activity (Itzkowitz 1984; Mackie and Itzkowitz 1985). In the presence of a former partner, female aggression may be displayed in order to reestablish these roles. However, as females are more commonly abandoned by males, they may be less receptive to a novel individual as this places their offspring and future reproductive success at risk.

Experimental resident males and females showed more aggression toward their partners compared with control resident males and females. However, experimental pairs did not show indications that their pair bond had broken apart, as this is usually accompanied by individuals suffering injuries to the body and fins and often leading to serious injury, or even death (T. Paciorek, personal observations)*.* Aggression between males and females may be based on whether or not individuals will try to attempt to reincorporate themselves with their former partner, and whether or not both members of the pair recognize their partner and the bond. In the Texas cichlid, pair bonds were shown to dissolve once pairs were separated for 4 or 10 days, and both males and females would attack their returned mate often to the point of injury or death (Mackie and Itzkowitz 1985)*.* However, the fish in this study were separated immediately after eggs were laid, and were not examined for parental care activities. Since males are expected to abandon pair bonds first (Balshine-Earn 1995), it is possible that they might show less interest in re-uniting with their partner and subsequently participating in parental care behaviors. However, if left as a resident to care for offspring, they may have increased their care behaviors to better ensure survival of offspring. It is possible that aggression may be displayed (particularly by resident fish) as a means of reaffirming roles and negotiating parental care duties within the pair bond. Theoretical investigations of parental care among monogamous systems suggest that parents may have set amounts, or “sealed bids” of investment they are willing to provide in a single reproductive bout (Houston and Davies 1995). This theory suggests that a parent will invest in offspring at a rate that provides it with the highest level of fitness possible, in relation to how much its partner invests. Thus, parental care may not necessarily be equal among pair bond members. Further models of parental investment have incorporated periods of time where partners may try to negotiate the amount of care they should provide as a pair (McNamara et al. 1999; Johnstone and Hinde 2006). As the needs of offspring will change over time, it allows both parents to make continuous decisions over how much care to provide. Aggression displayed by resident males to removed females was often executed by chasing females back to the clay pot shelter where the wrigglers were present, while aggression displayed by resident females to removed males would often result in females chasing males to the intruder cage and staying with the male momentarily to attack the intruder (T. Paciorek, personal observations). It is possible that due to greater investment in offspring development and direct offspring care that females may spend more time negotiating with males and adjusting their own behaviors as a way of maintaining stability within a pair bond.

Parental care behaviors were noticeably affected by periods of separation. Whereas females (regardless of group or status) spent more time with offspring compared with males, they showed a significant decrease in their time spent with offspring following the return of their partner. This may be explained by increases in aggression toward their partners. By spending more time interacting with males, presumably to try and reestablish sex-specific parental care roles, females reduce their own parental care roles. This is in opposition to the prediction that the resident individual would spend more time in both parental care roles to compensate for their partner. By leaving offspring alone, they are more susceptible to danger from predators. Males (regardless of group or status) spent more time interacting with the intruder compared with females. The primary parental care behavior of males is territorial defense (Alonso et al. 2001), and when isolated from partners are known to increase aggression as well as the amount of time spent near offspring (van Breukelen and Itzkowitz 2011)*.* However when reunited, experimental *A. nigrofasciata* pairs significantly reduced the amount of time spent interacting with the intruder. Similar to resident females, resident males may be spending more time trying to reestablish females in their offspring care roles than interacting with the intruder, resulting in reduced parental care. Offspring survival may diminish if parents are not fully partaking in parental care. It is still unknown what conditions could result in more immediate termination of pair bonds. Given that pairs were only observed after their initial reintroduction, it is possible that more long-term studies would provide further insight into whether pairs remain intact or show further signs of decline following separations. Furthermore, it is not entirely known whether removed males and females truly recognize their offspring following reintroductions, or if the presence of the resident fish was a deterrent to eating their offspring. Future experiments in which removed individuals are returned to their offspring without the resident may provide further information into their ability to recognize and care for offspring.

Resident fish cortisol levels were also examined before and after separations. Both control and experimental resident males showed no change in cortisol levels, while control and experimental resident females showed increased cortisol concentrations over time. The hypothesis that separation and reintroduction would increase levels of aggression and secretion of cortisol was supported, though increases were seen more prominently in females. While changes were apparent, it is worth noting that the inter-assay co-efficient of variance of each plate was high, which would require additional cortisol sampling in future experiments. Conflict among monogamous pairs is believed to result in periods of negotiation among individuals with regard to the amount of care that should be provided (McNamara et al. 1999). Any confusion over parental responsibilities may result in an increase in aggression on the part of the resident fish, which in turn could lead to increased cortisol circulation. The similarities in cortisol increases among control and experimental resident females (as well as increased aggression in the behavioral study) suggest that females consistently act to maintain male behavior within pair bonds. Given that females must invest in egg production, provide more direct offspring care, and are more likely to be abandoned by males at some point during the pair bond (Trivers 1972; Wisenden 1995), they may display these behaviors more strongly than males in order to maintain a pair bond and ensure offspring care.

In contrast to females, resident males (both control and experimental) showed little change in circulating cortisol levels. Paternal care and investment across taxa can be impacted by a variety of factors (for review, see Alonzo 2010), and studies involving parental care often focus on males, as paternal care is rarer across taxa (with the exception of birds), which usually involves manipulation of male behavior in order to limit their likelihood of providing adequate care (Hegner and Wingfield 1987). Males typically are more likely to abandon pair bonds compared with females (Balshine-Earn 1995; Wisenden et al. 2008), usually due to acquiring additional breeding opportunities or if situations, such as high resource availability, do not favor their input. Thus, unlike females, such parental care situations may not result in males displaying increased cortisol levels, as they may choose to invest less in offspring care and take advantage of other resource opportunities. In addition, teleosts typically do not engage in paternal only care. Such situations may result in males being less efficient in maintaining or establishing females in their sex-specific parental roles compared with females.

While cortisol increases have been linked to increased aggression in other species (Mikics et al. 2004; Earley et al. 2006), increases in both aggression and circulating cortisol levels in this study were not shown to be strongly correlated (males: *R*^2^ = 0.014; females: *R*^2^ = 0.0063). It is possible, however, that additional factors, both behavioral and hormonal, may be influencing these changes. The neuroendocrine stress response has many functions, including increasing metabolic energy available to respond to conflict with conspecifics, as well as adaptation to the environment through interactions with stimuli from interspecific and conspecific individuals (reviewed by Goymann and Wingfield 2004). The stress response is most often associated with the reaction to threat or danger, but also, the stress response is involved in social interactions, including social conflict, territorial display, and parental care. This pathway is a multi-armed array of neuroendocrine events, including the rapid (within seconds) sympathoadrenal response that leads to the secretion of epinephrine and norepinephrine, the slightly slower (within minutes) central nervous system secretion of neuropeptides with direct effects on brain and behavior (e.g., secretion of vasopressin and corticotropin-releasing hormone), and the relatively slower (minutes to hours) hypothalamic–pituitary–adrenal (HPA) response (reviewed by Chrousos 1998). The latter response leads to the secretion of the glucocorticoids, such as cortisol. The HPA axis is only one part of the neuroendocrine stress response, and it is likely that innervations in other regions of this pathway, such as activation of the sympathetic nervous system, may influence the release of other hormones. In addition, cortisol has a variety of roles that are activated in response to stress, including physiological changes such as metabolic resource distribution and maintenance of homeostatic state (reviewed by Chrousos 1998; Madison et al. 2018). Future examination of metabolic activity in stressed pairs may provide additional information into how various physiological states may influence changes in behavior, both between pair bond members and in relation to offspring care and vigilance with regard to returning partners. The relationship between cortisol and other circulating hormone levels requires further investigation to determine how their underlying functions may be impacting the maintenance of a pair bond as well as parental care behaviors.

A functioning pair bond is essential to the survival of offspring. However, when pairs are subject to conditions that may result in conflict, this has the potential to decrease their efficiency as parents and lead to the termination of a pair bond. This study demonstrated that while pair bonds appear to maintain stability following periods of separation, changes in behavior, specifically aggression directed toward investment in parental care roles, lead pairs to decrease offspring care. This study also demonstrated that only females showed consistent increases in cortisol across both treatment groups, while males did not. As such, males and females may differ in their responses to partner removal with regard to interactions that may maintain or establish parental care behaviors. Stress within and among pair bonded individuals has the potential to cause long lasting changes within monogamous mating systems. This research provides more information to the function and evolutionary significance of both monogamous mating systems and biparental care.
